# Agarolytic Pathway in the Newly Isolated *Aquimarina* sp. Bacterial Strain ERC-38 and Characterization of a Putative β-agarase

**DOI:** 10.1007/s10126-023-10206-7

**Published:** 2023-04-01

**Authors:** Ji Young Kang, Ha-Yeon Song, Jung-Mi Kim

**Affiliations:** 1 Industrial Microbiology and Bioprocess Research Center, Korea Research, Institute of Bioscience and Biotechnology (KRIBB) , Jeongeup, Jeonbuk 56212 Republic of Korea; 2 Department of Life and Environmental Sciences, Institute of Life Science and Natural Resources, Wonkwang University, Iksan, Jeonbuk 54538 Republic of Korea

**Keywords:** *Aquimarina* bacterium, Marine polysaccharides, Agarolytic pathway, Polysaccharide utilization loci (PULs), β-Agarase, Heterologous expression

## Abstract

Marine microbes, particularly Bacteroidetes, are a rich source of enzymes that can degrade diverse marine polysaccharides. *Aquimarina* sp. ERC-38, which belongs to the Bacteroidetes phylum, was isolated from seawater in South Korea. It showed agar-degrading activity and required an additional carbon source for growth on marine broth 2216. Here, the genome of the strain was sequenced to understand its agar degradation mechanism, and 3615 protein-coding sequences were predicted, which were assigned putative functions according to their annotated functional feature categories. In silico genome analysis revealed that the ERC-38 strain has several carrageenan-degrading enzymes but could not degrade carrageenan because it lacked genes encoding κ-carrageenanase and S1_19A type sulfatase. Moreover, the strain possesses multiple genes predicted to encode enzymes involved in agarose degradation, which are located in a polysaccharide utilization locus. Among the enzymes, Aq1840, which is closest to ZgAgaC within the glycoside hydrolase 16 family, was characterized using a recombinant enzyme expressed in *Escherichia coli* BL21 (DE3) cells. An enzyme assay revealed that recombinant Aq1840 mainly converts agarose to NA4. Moreover, recombinant Aq1840 could weakly hydrolyze A5 into A3 and NA2. These results showed that Aq1840 is involved in at least the initial agar degradation step prior to the metabolic pathway that uses agarose as a carbon source for growth of the strain. Thus, this enzyme can be applied to development and manufacturing industry for prebiotic and antioxidant food additive. Furthermore, our genome sequence analysis revealed that the strain is a potential resource for research on marine polysaccharide degradation mechanisms and carbon cycling.

**Supplementary Information:**

The online version contains supplementary material available at 10.1007/s10126-023-10206-7.

## Introduction

 Seaweed includes red, brown, and green algae and contains 50–70% carbohydrates based on its dry weight, including complex and diverse polysaccharides (e.g., agars, carrageenans, porphyrans, pectins, alginates, and fucoidans) (Percival 1979; Sudhakar et al. 2017; Morrice et al. 1984). Among them, agar comprises a well-known but complex cell wall polysaccharide family from marine red algae that in general can roughly be divided into the neutral “agarose” fraction and the charged “agaropectin*.*” Agarose is a linear polysaccharide mainly composed of basic alternating units of 3-O-linked-β-D-galactose and 4-O-linked 3,6-anhydro-α-L-galactose, and agaropectin comprises a similar galactose-based backbone but heterogeneously contains acidic side groups, such as sulfate and pyruvate.

Polysaccharide biolysis is a key step in the carbon cycle of marine ecosystems (Imran et al. 2017). Agarase is the first key enzyme in the agar-degrading pathway that catalyzes the hydrolysis of agar into agaro-oligosaccharides (AOSs) or neoagaro-oligosaccharides (NAOSs). Agarase is classified into α-agarase and β-agarase based on the cleavage mechanism; α-agarase cleaves the α-1,3-glycosidic linkage to generate AOSs, and β-agarase cleaves the β-1,4-glycosidic linkage to generate NAOSs. α-agarases belong to the GH96 group of the glycoside hydrolase (GH) family, and β-agarases belong to the GH16, GH50, GH86, and GH118 groups (Fu and Kim 2010; Chi et al. 2012; Jagtap and Manohar 2021).

Numerous marine microbes have enzymes that degrade polysaccharides, including agar (Arnosti 2011). Bacteroidetes*,* which is a very diverse bacterial phylum, generally have a greater enzymatic potential for carbohydrate degradation than other bacterial phyla. These carbohydrate-active enzymes often cluster in polysaccharide utilization loci (PULs), which by definition have capacities for sensing, degradation, transport, and metabolism of a given polysaccharide (Lapébie et al. 2019; Grondin et al. 2017). In bacterial cells, polysaccharide-degrading enzymes primarily exist in the periplasmic space and catalyze the breakdown of polysaccharides in the periplasm that are imported by transporters encoded by the *susC/D* gene pair. The oligomers generated by polysaccharide breakdown can be subsequently degraded by sugar-cleaving enzymes (Lapébie et al. 2019).

Here, we report the complete genome sequence of a marine bacterium, ERC-38, that exhibited a concave surface around colonies on an agar medium. The 16S rRNA gene sequence of the ERC-38 strain indicated that it belongs to the genus *Aquimarina* and is a potential novel species. To identify possible agarolytic pathways in the ERC-38 strain, the molecular and enzymatic characteristics of its agarolytic enzymes were investigated. This study provides additional information about algal biomass decomposition and recycling by algal polysaccharide-degrading bacteria. Aq1840 agarase reported in this study also has potential for the production of bioactive algal oligosaccharides.

## Materials and Methods

### Bacterial Strains and Culture Conditions

A seawater sample collected from the Yellow Sea coast (Buan-gun), South Korea, was diluted with sterilized seawater and spread onto marine broth 2216 (Difco) containing 2% agar (MA) plates. The strain was routinely cultured on MA plates and maintained at − 80 °C with 20% (v/v) glycerol. To examine carbon source utilization, the strain was pre-grown in marine broth containing 0.5% glucose, inoculated into marine medium containing 0.1–1% glucose, galactose, agarose (low melting agarose, Duchefa), κ-carrageenan (Sigma) or ι-carrageenan (Sigma), and incubated at 25 °C for 3 days. To determine polysaccharide degradation ability using a plate assay, the pre-grown cells were inoculated on a plate containing marine broth diluted 10-time supplemented with 3.6% sea salt (Sigma), and 2% agar (Bacto™ Agar, Difco), κ-carrageenan or ι-carrageenan as the carbon source and incubated at 25 °C for 4 days. The degradation ability was determined by colony growth and iodine staining.

### Phylogenetic Analysis and Whole-Genome Sequencing

The 16S rRNA gene was amplified from purified genomic DNA using universal primers (27F and 1492R). The pairwise gene similarity of 16S rRNA was determined using the EzBioCloud database (https://www.ezbiocloud.net/, (Yoon et al. 2017)) and the BLAST program. Phylogenetic tree was conducted using MEGA X after performing multiple alignments of the sequences and constructed with neighbor-joining (NJ) method (Kumar et al. 2018; Saitou and Nei 1987).

For whole-genome sequencing of the ERC-38 strain, genomic DNA was extracted from cells using a genomic DNA purification kit (Invitrogen), according to the method provided by the manufacturer. Sequencing and assembly method is presented in Table 1. Circular genome was drawn using the CGView program (Grant and Stothard 2008). DNA-DNA hybridization (DDH) and the average amino acid identities (AAIs) were calculated using a method described in previous studies (Kang et al. 2022) and the color-coded heatmap was generated using CIMminer software (http://discover.nci.nih.gov/cimminer).

**Table 1:** General features and genome sequencing project information for *Aquimarina* sp. ERC-38

**Items**	**Description**
**General features**	
Classification	Bacteroidetes; flavobacteriia; flavobacteriales; flavobacteriaceae; aquimarina
Strain	ERC-38
Latitude and longitude	35°77′N, 126°57′E
Geographical location	Yellow Sea, South Korea
Environment	Seawater
Collection date	Oct-2019
Oxygen requirement	Aerobic
**Genome characteristics**	
Sequencing platform	PacBio RSII
Assembly method	HGPA 3.0
Genome coverage	212.4x
Finishing strategy	Complete
BioProject	PRJNA843789
BioSample	SAMN28771315
**Genomic features**	
NCBI accession number	CP098511
Size (bp)	4,431,522
DNA G + C content (%)	34.8
CDSs	3,615
tRNAs	40
rRNAs	9
**Summary of CAZymes**	
Glycoside hydrolases (GHs)	65
Glycosyl transferases (GTs)	62
Polysaccharide lyases (PLs)	6
Carbohydrate esterases (CEs)	6
Auxiliary activities (AAs)	2
Carbohydrate-binding modules (CBMs)	11

### Sequence Analysis of the Agar-degrading Enzyme Genes

Genes involved in polysaccharide metabolism were identified based on annotations in the Swiss-Prot and Carbohydrate-Active Enzyme (CAZy) databases. The molecular weight and predicted structure of the proteins were estimated using the pI/Mw or modeling tools on the ExPASy server of the Swiss Institute of Bioinformatics (Gasteiger et al. 2005). The presence of a signal sequence was predicted using the SignalP 5.0 server (Almagro Armenteros et al. 2019), and the conserved domain was detected using the NCBI’s conserved domain database (CDD). The conserved domain was reconfirmed by comparison with characterized GH16 family members in the CAZy database. Multiple alignment of the amino acid sequences of agarases was constructed using the Clustal_W algorithm in MEGA X as described above.

### Cloning and Expression of the β-Agarase Gene

The mature sequences of agarase genes were amplified from the genomic DNA of the ERC-38 strain using primer sets (Table S1). Each of the amplified fragments were inserted into the pET21a vector using the AccuRapid Cloning kit (Bioneer Inc.) to generate expression vector, pET21a-Aq1828, -Aq1829, -Aq1830, -Aq1832, -Aq1839, or -Aq1840. After confirmation of the accuracy of the sequences, each expression vector was transformed into *Escherichia coli* BL21 (DE3) codon + competent cells.

The transformed *E. coli* BL21 (DE3) codon + cells were cultured in LB medium containing ampicillin (100 μg/mL) (LA). To induce gene expression, the transformed cells were pre-grown at 37 °C for 14 h and transferred to new LA medium containing 0.5 mM IPTG, and incubated for 6 h at 37 °C. Crude enzymes were obtained from the gene induced cells by disruption using sonication under a resuspension buffer (40-mM Tris–HCl buffer (pH 8.0), supplemented with 50 mM NaCl and 1 mM dithiothreitol (DTT)). Protein expression was detected by sodium dodecyl sulfate–polyacrylamide gel electrophoresis (SDS-PAGE), and the gels were stained with Coomassie Brilliant Blue R-250 (Bio-Rad).

### Purification of Recombinant Aq1840 β-Agarase

To purify the recombinant enzyme, the crude enzyme solution was applied to a HisTrap HP column (GE Healthcare) using an FPLC system (ÄKTA Pure 25, GE Healthcare). The enzyme sample was washed with three volumes of washing buffer (40 mM Tris–HCl, 500 mM NaCl, and 20 mM imidazole, pH 8.0) and eluted with an elution buffer (40 mM Tris–HCl, 500 mM NaCl, and 500 mM imidazole, pH 8.0). The eluted enzyme was concentrated using an Amicon Ultra-15 Centrifugal Filter Unit with an Ultracel-3 membrane (EMD Millipore) for desalting and resuspended in a 40-mM Tris–HCl buffer (pH 8.0) supplemented with 1 mM DTT. The enzyme solution was used to measure agarase activity.

### Enzyme Activity Assay and Biochemical Characterization

For the enzymatic reaction, 100 μL of the purified enzyme solution was mixed with an equal volume of 1.5% (w/v) agarose dissolved in a 40-mM Tris–HCl buffer containing 1 mM DTT (pH 8.0). Temperature tests were performed with the mixtures in a temperature range of 30–50 °C (5 °C intervals) after incubation for 3 h. pH tests were performed in a range of 5.5–9.0 (0.5 unit intervals) using a McIlvaine buffer (pH 5.5–7) or a Tris–HCl buffer (pH 7.5–9.0). Thermostability of the enzyme was investigated by pre-incubating the enzyme at 30–60 °C for 3 h and then analyzing the residual activity at 40 °C for 3 h with equal volume of 1.5% (w/v) agarose. The effects of metal ions on enzyme activity were assayed using a buffer supplemented with various ions (10 mM). Quantification of agarase activity using the previously described enzyme reaction conditions was performed using the 3,5-dinitrosalicylic acid method, which detects reducing sugars released from agarose.

### Identification of Enzyme Reaction Products by Thin-layer Chromatography

Hydrolysis reactions using purified recombinant Aq1840 were performed at 40 °C with 0.75% (w/v) agarose in a 40 mM-Tris–HCl buffer containing 30 mM NaCl and 1 mM DTT (pH 8.0), and the reaction time was varied from 15 min to 72 h. To identify the minimum hydrolysis substrate, 1% AOSs and NAOSs (Biosynth, Ltd.) were included as the only substrate using the method mentioned above.

TLC was performed using silica gel 60 aluminum plates (F254 Merck) to detect the hydrolytic products, which were developed using n-butanol-acetic acid–water (2:1:1, v/v). Saccharides were detected using a visualization solution containing 10% (v/v) sulfuric acid in ethanol, followed by heating at 100 °C.

## Results

### Isolation and Identification of the Agar-degrading Bacterium ERC-38

To understand the diverse degradation mechanisms of marine polysaccharides by marine bacteria, various bacteria were isolated from seawater. Among these, we found that one bacterium generated a concave surface on the agar medium (Fig. S1a). Because this phenotype indicated that the strain could degrade agar, the isolate was selected to examine the diversity of hydrolytic enzymes present in the strain.

To assess the agar-decomposing ability of the ERC-38 strain, we performed a plate assay that visualized this ability using iodine staining. In agar-containing plates, the strain grew with a concave surface and transparent zone around the colonies (Fig. S1a). Interestingly, the isolate grew well in agar-containing marine medium but did not grow in a standard marine broth. The isolate grew in marine broth supplemented with carbon sources such as glucose, galactose, or agarose (Fig. S1b), which implies that the isolate requires a sufficient carbon source for growth.

**Fig. 1:**
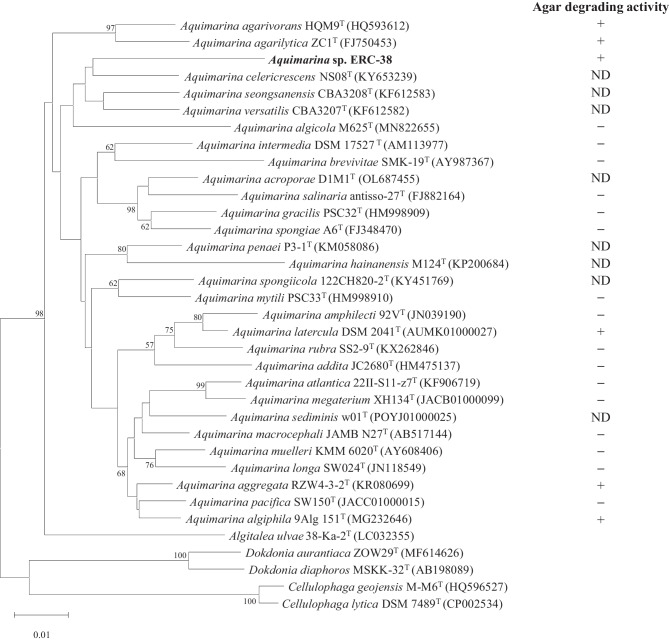
Neighbor-joining tree, based on 16S rRNA gene sequences (1,451 bp), showing the phylogenetic relationships of the ERC-38 strain and 29 species belonging to the *Aquimarina* genus*.* Bootstrap percentages are based on 1000 replicates; only values greater than 50% are shown at the nodes. Bar, 0.01 substitutions per nucleotide position. For agar-degrading activity, + indicates the presence of activity, − indicates a lack of activity, and ND indicates not determined in the previous study

To identify the taxonomic location of the strain, the 16S rRNA gene sequence was amplified from the genome of the isolate and sequenced. A comparative analysis of the 16S rRNA gene using the EzBioCloud database showed that a 1451-bp continuous sequence of the ERC-38 strain was most similar to that of *Aquimarina celericrescens* NS08^T^, with a sequence similarity of 95.2%. The ERC-38 strain also showed 92.6–94.9% sequence similarity with other members of the *Aquimarina* genus, containing 29 recognized species (LPSN, List of Prokaryotic names with Standing in Nomenclature), including the recently published species *A. acroporae* D1M1^T^ (Sun et al. 2022). In phylogenetic analysis based on the 16S rRNA gene, ERC-38 comprised a clade with *A. celericrescens* NS08^T^ within the genus *Aquimarina,* belonging to the family *Flavobacteriaceae* (Fig. 1).

### Genomic Sequence Analysis of Bacterium ERC-38

**Fig. 2:**
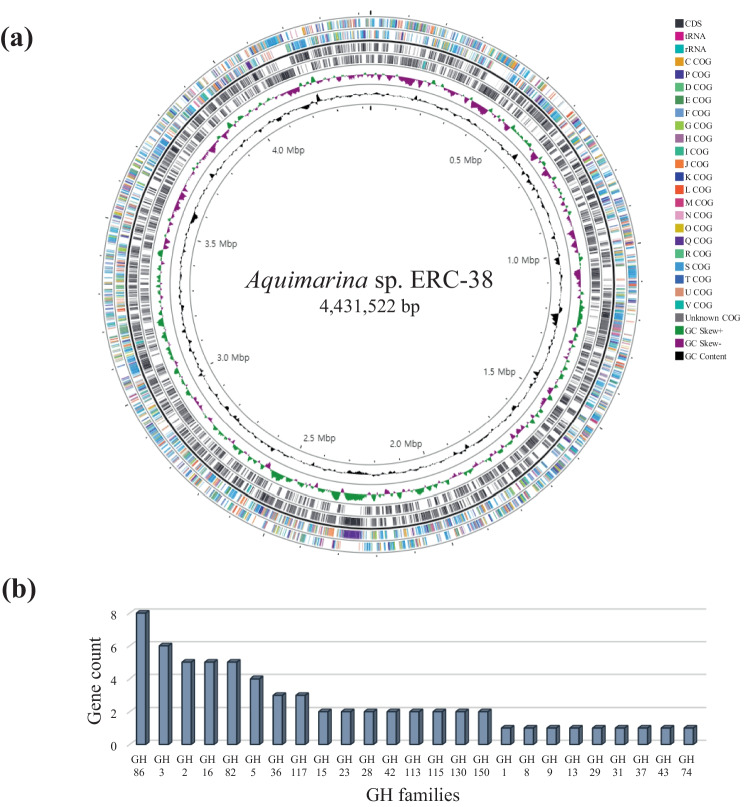
Circular genome diagram and carbohydrate-active enzymes predicted to be encoded in the *Aquimarina* sp. ERC-38 genome. **a** Schematic representation of the genome. Labeling, from the center to outside, is as follows: circle 1, scale line in Mbps; circle 2, regions with a GC percentage higher or lower than the average value are shown outside or inside the center; circle 3, GC skew index according to the formula, (G − C)/(G + C), showing a higher G content (in green) and a higher C content (purple peaks); circles 4 and 5, CDS/tRNA/rRNA genes; circles 6 and 7, CDSs colored according to COG function categories. **b** Gene counts of GHs corresponding to the CAZy functional classification

Genome sequence data of the ERC-38 strain have been submitted to the GenBank database under accession number CP098511. Whole-genome sequencing showed that the ERC-38 strain has a circular chromosome consisting of 4,431,522 bp with a GC content of 34.8 mol% and no plasmid (Fig. 2a). Genome annotation using the PGAP revealed that the genome of strain ERC-38 was predicted to encode 3615 coding sequences (CDSs), 40 tRNAs, and 9 rRNAs (Table 1). The majority of the CDSs (3,381/3,685; 91.8%) could be assigned a putative function according to their annotated functional feature (Clusters of Orthologous Groups, COG) categories (Fig. 2a).

According to the CAZy database, the ERC-38 strain genome was predicted to encode 65 GHs, 62 glycosyl transferases, 6 polysaccharide lyases, 6 carbohydrate esterases, 2 auxiliary activities, and 11 carbohydrate-binding modules (Table 1). Within the GHs, GH86 was the most abundant family in the genome, followed by GH3, GH2, GH16, and GH82 (Fig. 2b).

**Fig. 3:**
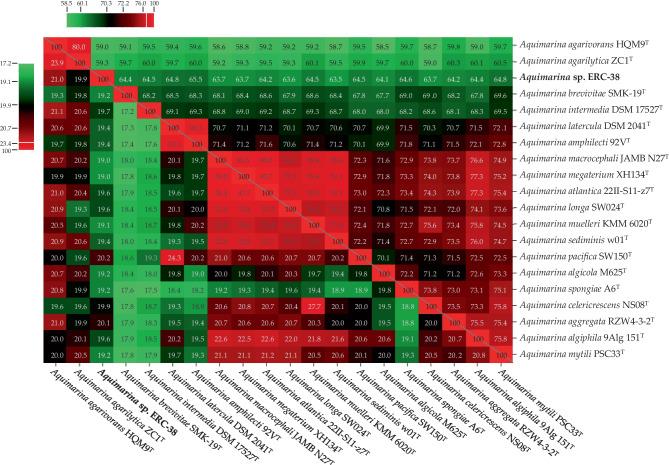
Heat map of DDH and average AAI values of genomes of the isolate and species belonging to the *Aquimarina* genus. The lower triangle displays the DDH values, and the upper triangle displays the AAI values. Isolates and related strains are given as row and column labels on the map. Green, black, and red cells correspond to low, middle, and high values, respectively, as indicated by color bars to the left and above the heat map

The heat map results for the percentage of genome and protein similarities between ERC-38 and other species belonging to the *Aquimarina* genus are presented in Fig. 3. The in silico DDH and AAI values ranged from 19.0 to 21.0 and from 59.0 to 65.5, respectively. Although the DDH and AAI values of ERC-38 were somewhat lower than those of other strains, they were within the range of values of all strains of the *Aquimarina* genus (17.2–47.7 and 58.5–93.7, respectively; Fig. 3).

### Agarolytic System Genes and Analysis of Agarase Enzymes

**Fig. 4:**
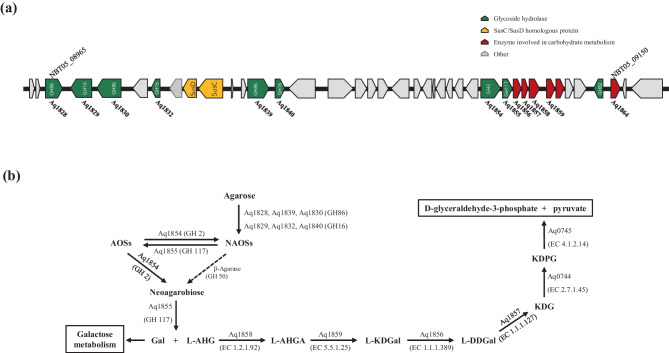
Genes of the PUL-a and tentative pathway involved in the agarose metabolism in the ERC-38 genome. **a** Each arrow indicates a separate gene, and the size is proportional to the gene length. Predicted functions are color-coded and presented above the arrows. **b** Enzymes are indicated at each reaction step along with the CAZy family or EC number. Solid arrows represent the proposed pathway for ERC-38 based on functional and bioinformatic analyses and dotted arrow represents an absent pathway. NAOSs, neoagaro-oligosaccharides; AOSs, agaro-oligosaccharides; Gal, D-galactose; L-AHG, 3,6-anhydrogalactose; L-AHGA, 3,6-anhydro-L-galactonate; L-KDGal, 2-keto-3-deoxy-L-galactonate; L-DDGal, 2,5-diketo-3-deoxy-L-galactonate; KDG, 2-keto-3-deoxy-D-gluconate; KDPG, 2-keto-3-deoxy-6-phospho-D-gluconate

In general, endolytic agarases catalyze the initial step of agarose degradation. In the genome of the strain, six genes encoding putative GH16 or GH86 β-agarases were co-localized with SusD/TonB transporters in a PUL designated PUL-a (locus tag, NBT05_08965 ~ 09,150) (Fig. 4a). PUL-a also contained β-galactosidase (GH2) and α-neoagaro-oligosaccharide hydrolase (GH117), which participate in agarose degradation. Agarose is hydrolyzed into NAOSs by endo-acting GH16 and/or GH86 β-agarases and is then hydrolyzed by exo-acting GH2 and GH117 family proteins into monomers, D-galactose, and 3,6-anhydrogalactose. Moreover, based on the sequence analysis and comparison with functionally validated enzymes, we identified all genes involved in the agarose metabolic pathway (Fig. 4b; Table 2). Four genes encoding putative enzymes including L-AHG dehydrogenase (EC 1.2.1.92), L-AHG cycloisomerase (EC 5.5.1.25), 2-keto-3-deoxy-L-galactonate 5-dehydrogenase (EC 1.1.1.389), and 2,5-diketo-3-deoxy-L-galactonate 5-dehydrogenase (EC 1.1.1.127) were localized in the PUL-a (Fig. 4a). Two genes predicted to encode 2-keto-3-deoxy-D-gluconate kinase (EC 2.7.1.45) and 2-keto-3-deoxy-(6-phospho)-gluconate aldolase (EC 4.1.2.14), which are involved in this pathway were found in another location (locus tag, NBT05_03640 and NBT05_03645).

**Table 2:** Putative enzymes in the agarolytic PUL region involved in agarose metabolic pathway of *Aquimarina* sp. ERC-38

**Protein**	**Locus tag**	**Closest characterized protein**	**Accession no**	**CAZy family/EC no**	**Percent identity**
Aq0744	NBT05_03640	2-Keto-3-deoxygluconate kinase	2AFB_A	EC 2.7.1.45	37.5%
Aq0745	NBT05_03645	2-Keto-3-deoxy-6-phosphogluconate aldolase	8DI1_A	EC 4.1.2.14	41.5%
Aq1828	NBT05_08965	β-Agarase	5TA1_A	GH86	38.9%
Aq1289	NBT05_08970	β-Agarase D	4ASM_B	GH16	47.9%
Aq1830	NBT05_08975	β-Agarase	5TA1_A	GH86	29.2%
Aq1832	NBT05_08985	β-Agarase	3WZ1_A	GH16	33.6%
Aq1839	NBT05_09015	β-Agarase	5TA1_A	GH86	45.5%
Aq1840	NBT05_09020	β-Agarase C	6HY3_A	GH16	61.6%
Aq1854	NBT05_09100	β-Aalactosidase	5T9A_A	GH2	53.3%
Aq1855	NBT05_09105	3,6-Anhydro-alpha-L-galactosidase	3P2N_A	GH117	69.5%
Aq1856	NBT05_09110	2-Dehydro-3-deoxy-L-galactonate 5-dehydrogenase	1E3J_A	EC 1.1.1.389	31.2%
Aq1857	NBT05_09115	2-Dehydro-3-deoxy-D-gluconate 5-dehydrogenase	4Z9Y_A	EC 1.1.1.127	47.5%
Aq1858	NBT05_09120	3,6-Anhydro-alpha-L-galactose dehydrogenase	6J75_A	EC 1.2.1.92	69.3%
Aq1859	NBT05_09125	3,6-Anhydro-L-galactonate cycloisomerase	5XD7_A	EC 5.5.1.25	69.1%

Each β-agarase, Aq1828, Aq1829, Aq1830, Aq1832, Aq1839, and Aq1840, had a signal peptide at the N-terminal. When the proteins were compared against the PDB database using NCBI BLAST, Aq1828, Aq1830, and Aq1839 were found to have a GH86 catalytic domain, and the amino acid sequences of the β-agarases shared the highest sequence identity (38.9%, 29.2%, and 45.5%, respectively) with the β-agarase of *Bacteroides uniformis* (accession no. 5TA1_A; Pluvinage et al. 2018). Aq1829 and Aq1840 had a GH16 catalytic domain, and the amino acid sequences of the β-agarases shared the highest sequence identity with β-agarase D (47.9%, accession no. 4ASM_B; Hehemann et al. 2012) and β-agarase C (61.6%, accession no. 6HY3_A; Naretto et al. 2019) of *Zobellia galactanivorans*. Aq1832 also had a GH16 catalytic domain and showed the highest sequence identity (33.6%) with β-agarase (accession no. 3WZ1_A; Takagi et al. 2015) of *Microbulbifer thermotolerans*. Further analysis of the phylogenetic relationship based on the amino acid sequences of characterized enzymes in the CAZy database also showed that Aq1828, Aq1830, and Aq1839 belonged to the GH86 family and Aq1829, Aq1832, and Aq1840 belonged to the GH16 family (Fig. S4).

### Expression and Activity Test of Recombinant 1840 Agarases

To examine the properties of the six putative β-agarases, the genes were cloned and expressed in *E. coli* BL21 (DE3) codon + strains. However, only recombinant Aq1840 was presented in the soluble fraction together with agar-degrading activity, and other recombinant enzymes were present in the cell lysate precipitate as inclusion bodies (Fig. S5).

**Fig. 5:**
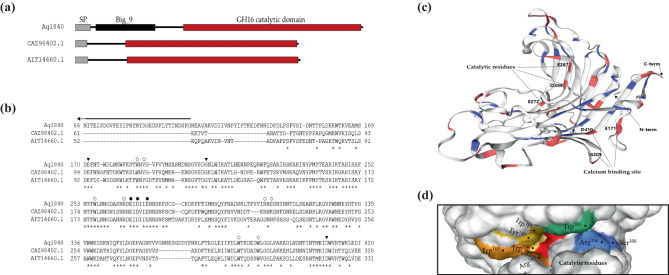
Structure and multiple alignments of the amino acid sequence of the Aq1840 agarase of ERC-38. Linear structure (**a**) and sequence alignment (**b**) of Aq1840 and other β-agarases. **a** Signal peptide (SP), bacterial immunoglobulin class 9 (Big_9) domain, and GH16 catalytic domain are represented as gray, black and red boxes, respectively. **b** Identical residues in all sequences are indicated by asterisks under the column. Conserved residues involved in substrate recognition are indicated by open circles. Catalytic or metal ion-binding residues are highlighted by closed circles or triangles, respectively. The Big_9 domain is indicated by a line above the sequences. CAZ98402.1, β-agarase of *Zobellia galactanivorans*; AIT14660.1, β-agarase of *Catenovulum agarivorans* YM01. **c** In the 3D structure, blue, red, and white represent basic, acidic, and other amino acid residues, respectively. Catalytic or metal ion-binding residues are indicated in the structure. **d** Predicted residues involved in substrate recognition are indicated in the structure as colored in the 3D structure. The amino acids involved in substrate recognition are colored as follows: subsite − 2 or − 3, orange; subsite − 1, yellow; subsite + 1, blue; subsite + 2, green; catalytic residues, red

Aq1840, the only successfully expressed β-agarase in this study, includes an open reading frame consisting of 1263 nucleotides, encoding a 421-amino acid protein, and its predicted molecular mass is 46.3 kDa. Aq1840 contains a signal peptide (1–20 amino acids), a bacterial immunoglobulin (Ig) domain (31–118 amino acids) belonging to the Big_9 superfamily, and a GH16 catalytic module (160–416 amino acids) (Fig. 5a). In the amino acid sequence alignment of relative β-agarases, Aq1840 showed sequence similarity with other β-agarases, but the Ig domain was only present in Aq1840 (Fig. 5a and b). The conserved active site residues and ion-binding sites were represented in a three-dimensional (3D) structure simulation of the protein. The predicted substrate catalytic residues were Glu267, Asp269, and Glu272, and the predicted ion-binding site residues were Glu171, Gly205, and Asp410 (Fig. 5c). A previous study reported that ZgAgaC, closest homologue of Aq1840, is highly divergent from characterized GH16 β-agarases (Naretto et al. 2019). As a result of comparison with ZgAgaC, the following conserved residues of Aq1840 were predicted to be involved in substrate recognition: Trp185 (subsite − 2 or − 3); Arg265 (subsite − 2); Tyr187, Trp256, and Trp383 (subsite − 1); Arg306 and Ser308 (subsite + 1); and Trp389 (subsite + 2) (Fig. 5d).

### Biochemical Characterization and Enzyme Activity of Aq1840 Agarase

**Fig. 6:**
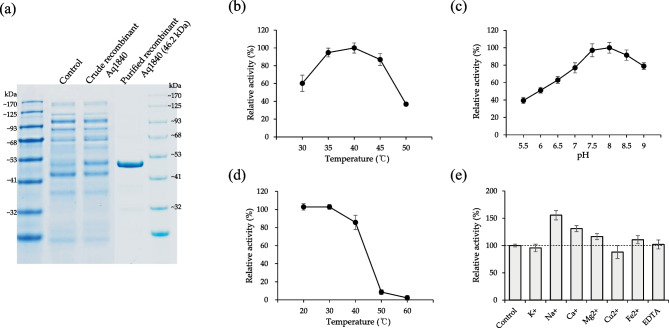
SDS-PAGE image and biochemical characteristics of purified recombinant Aq1840. **a** Recombinant Aq1840 was purified using a HisTrap column, and its purity was verified by SDS-PAGE analysis. Control or Crude Aq1840 indicates the soluble cell lysate obtained from an *E. coli* transformant containing an empty or a pET21a-Aq1840 vector, respectively. Purified Aq1840 indicates recombinant His-tagged Aq1840 purified from soluble cell lysate using a HisTrap column. **b** and **c** The effects of temperature and pH on the enzyme activity were measured using 0.75% agarose and the purified recombinant enzyme. **d** Temperature stability was investigated following a 3-h incubation at the indicated temperature. **e** The effects on the enzyme activity of 10-mM metal ion or EDTA (chelating agent) supplementation were compared with those in a non-treated control

To characterize recombinant Aq1840, protein purification was performed using Ni–NTA resin. Purified recombinant Aq1840 with His-tag showed a single band with a molecular weight of approximately 46.2 kDa on SDS-PAGE analysis (Fig. 6a). To characterize the enzymatic activity of recombinant Aq1840, we investigated various enzymatic factors such as temperature, pH, effects of metal ions, and temperature stability. Purified recombinant Aq1840 was examined at various temperatures (30–50 °C, 5 °C intervals), and the highest enzymatic activity was found at 40 °C. The optimal pH was 8 at 40 °C. More than 80% of the maximum activity was retained throughout the temperature range of 20–40 °C. When the pH was above 9 or below 7, recombinant Aq1840 showed less than 80% of its maximum activity (Fig. 6b–d). The presence of Na^+^ and Ca^2+^ ions enhanced the activity of recombinant Aq1840 by 1.56-fold and 1.31-fold, respectively, compared with that in the absence of ions (Fig. 6e). This result indicates that the ion-binding site regulates the conformational equilibrium and catalytic activity of the Aq1840 β-agarase by binding with Na^+^ or Ca^2+^ ions.

### Identification of Hydrolytic Products and Minimum Hydrolysis Substrate of Aq1840

**Fig. 7:**
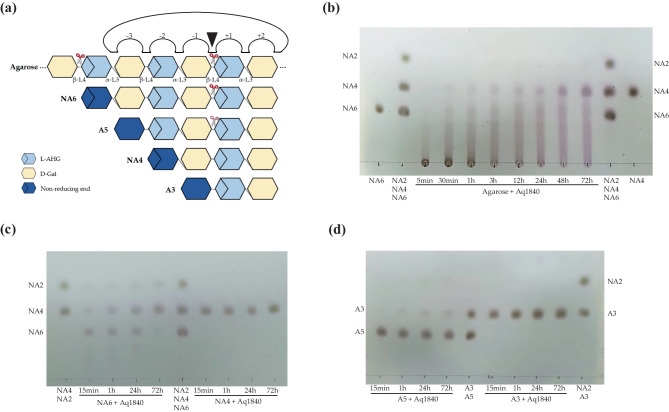
Time-kinetic analysis of Aq1840-catalyzed hydrolysates of agarose, NAOSs, and AOSs according to TLC. **a** Schematic model of the sub-binding sites of Aq1840 and enzymatic hydrolysis of agarose, NAOSs, and AOSs. Recombinant Aq1840 can produce NA4 and NA2 from NA6 and weakly produce A3 and NA2 from A5. **b**–**d** For hydrolysis of each substrate, 0.75% agarose (**b**), 1% NAOSs (**c**), and 1% AOSs (**d**) were incubated with purified recombinant Aq1840 in a 40-mM Tris–HCl buffer containing 30 mM NaCl and 1 mM DTT (pH 8.0) at 40 °C for the indicated time. The samples were visualized by spraying with an ethanol solution containing 10% (v/v) H_2_SO_4_ and then heating to 100 °C

To identify the final hydrolyzed agarose products generated by the Aq1840 enzyme, we performed TLC analysis of these hydrolysis products after time-kinetic treatment of agarose with purified recombinant Aq1840. Because Aq1840 showed homology with GH16 family proteins, it is speculated to be an endo-type enzyme and degrade agarose into NAOS with 4–10° of polymerizations (DPs). As a result of TLC analysis, the degradation model of Aq1840 is shown in Fig. 7a. Similar to other GH16 family β-agarases, Aq1840 showed endo-typic hydrolysis of agarose from the initiation of the reaction, and the final hydrolyzed product mainly consisted of NA4 (Fig. 7b). When NA6 and NA4 were used as substrates to investigate the minimum hydrolysis substrates of recombinant Aq1840, the recombinant enzyme eventually hydrolyzed NA6 to yield NA4 and NA2, but NA4 was not hydrolyzed (Fig. 7c). When the agaropentaose (A5) or agarotriose (A3) was used as the substrate, recombinant Aq1840 weakly hydrolyzed A5 to yield A3 and NA2, but it did not hydrolyze A3 (Fig. 7d). These results show that recombinant Aq1840 cleaves the β-1,4-linkage between A3 and NA2, which consistent with the mechanism of substrate recognition of ZgAgaC (Fig. 7a) (Naretto et al. 2019).

## Discussion

The previous result of statistical and clustering analyses of the marine heterotrophic bacteria shows that their carbohydrate catabolism correlates with both taxonomy and habitat (Barbeyron et al. 2016b). The genomes of marine bacteria reportedly contain genes encoding CAZymes for the degradation of marine polysaccharides (Hehemann et al. 2012; Boncan et al. 2018; Schultz-Johansen et al. 2018; Valdehuesa et al. 2018). Studies of the polysaccharide-degrading abilities and polysaccharide hydrolase characteristics of bacteria revealed their polysaccharide-metabolizing pathway and suggested that that pathway may be involved in the carbon cycle and biomass utilization in marine ecosystems (Imran et al. 2017; Valdehuesa et al. 2018; Gao et al. 2019). The marine bacterium ERC-38 strain grew well in marine broth supplemented with carbon sources representing abundant sugars of seaweed, such as glucose, galactose, or agarose (Fig. S1b). However, the isolate did not grow in medium containing another marine polysaccharide, carrageenan, as the carbon source (data not shown). This result implied that the isolate could not hydrolyze carrageenan and could not utilize it as a carbon source. Interestingly, the isolate could not grow on medium containing carrageenan as the carbon source but had several genes encoding enzymes involved in carrageenan degradation in a PUL (PUL-c; locus tag, NBT05_06965 ~ 07,125), which is including two putative GH150 family proteins (Aq1437 and Aq1450), and two GH82 family proteins (Aq1435 and Aq1440) (Fig. S2). According to the tentative carrageenan metabolic pathway, putative genes encoding enzymes such as α-1,3-(3,6)-anhydro-D-galactosidase (GH127, Aq0699), galactose mutarotase (Aq1864, in the PUL-a), galactokinase (Aq1452), galactose-1-phosphate uridylyltransferase (Aq1451), and UDP-galactose epimerase (Aq0608) were also found in the PUL-c or other loci of the genome (Fig. S3). Moreover, 17 putative S1-type sulfatases were also identified by a blast search on the sulfatase database server SulfAtlas (https://sulfatlas.sb-roscoff.fr/) (Stam et al. 2023), and these enzymes have been reported to be important for marine microbes to utilize highly sulfated algal polysaccharides (Barbeyron et al. 2016a; Hettle et al. 2018). Of these sulfatases, Aq2073 (locus tag, NBT05_10190), identified as S1_19, was classified as an S1_19B-type sulfatase and had the highest homology (57.6%) with PsS1_19B (6PRM_A) of *Pseudoalteromonas fuliginea* when the proteins were compared against Protein Data Bank proteins (PDB) using NCBI BLAST. S1_19B is an exo-G4S κ-carrageenan sulfatase that has κ-NC2 and κ-NC4 activity but does not have ι-NC2 or ι-NC4 activity (Hettle et al. 2019). The other 14 putative sulfatase genes, except for Aq2073 (S1_19), Aq3091 (S1_16), and Aq3095 (S1_16), were concentrated in a sizeable PUL containing the PUL-c (Fig. S2). These in silico data explain why the isolate could not grow when carrageenan was the carbon source. Although the isolate had many genes involved in carrageenan degradation and metabolism, it is assumed that carrageenan could not be completely hydrolyzed to neocarrabiose; ι-carrageenan oligosaccharides were produced from ι-carrageenan by Aq1435, Aq1440, or Aq1863, but desulfation could not occur. Additionally, exo-G4S κ-carrageenan sulfatase was present, but κ-carrageenase, which is used to generate κ-carrageenan oligosaccharides, was absent in the genome (Fig. S3). Therefore, further study is needed to verify this pathway.

Unlike its inability to degrade carrageenan, the isolate could degrade agarose. Therefore, we also investigated the agarose degradation process of the isolate at the genomic and enzymatic levels. Several species of the genus *Aquimarina*, as shown by their species name, including *A. agarivorans* HQM9^T^, *A. agarilytica* ZC1^T^, and *A. aggregata* RZW4-3-2^ T^, exhibit agar-degrading activity (Zhou et al. 2015; Lin et al. 2012; Wang et al. 2016). Among 29 species of the *Aquimarina* genus, five species including *A. latercula* DSM2041^T^ and *A. algiphila* 9Alg 151^ T^ as well as the above-mentioned three species have demonstrated agar-decomposing ability. Sixteen species do not have this ability, and another eight species have not been evaluated at this time (Fig. 1) (Nedashkovskaya et al. 2018; Li et al. 2014; Han et al. 2017; Park et al. 2013; Chen et al. 2012; Sun et al. 2021; Zhou et al. 2015; Wang et al. 2016; Lin et al. 2012). Although several species belonging to the *Aquimarina* genus have agar-degrading ability, only enzymatic characteristics of Aga672 and Aga575 of *A. agarilytica* ZC1^T^ have currently been reported. Aga672 is a GH16 β-agarase, and its final hydrolyzed products are NA4, NA6, and NA8, and Aga575 is an exo-type agarase and generates only NA2 as a final product (Dong et al. 2021; Lin et al. 2017). Besides, the production of neoagarooligosaccharides using a β-agarase of *A. agarilytica* NI125 has been reported, but the classification of the enzyme and the characteristics of the hydrolyzed products have not been specified (Farahat 2019).

For characterization of the β-agarases of the isolate, the six putative genes were expressed together with the addition of a His_6_ tag in *E. coli* BL21 (DE3) codon + strains. In a plate assay performed to detect agar-degrading activity, lysates of cells expressing recombinant Aq1829 and Aq1840 showed activity after iodine staining, but other putative enzymes did not show activity (Fig. S5a). SDS-PAGE showed that all expressed enzymes except recombinant Aq1840 were present in the cell lysate precipitate as inclusion bodies (Fig. S5b). Because the recombinant Aq1829 lysates exhibited activity in the plate assay, we attempted to obtain recombinant Aq1829 protein in a soluble state. However, it was insufficiently expressed as a soluble protein, and the precipitated protein lost activity after renaturation (data not shown).

In the amino acid sequence alignment, the Ig domain was presented in Aq1840 but not in other relative β-agarases. Ig-like domains, which have been previously shown to be present in bacteriophage and cell–cell adhesion proteins, are also distributed in a variety of GHs. However, the function of Ig domains is still not well understood, but it is speculated that these domains facilitate enzymatic function (Xing et al. 2022).

In TLC analysis, Aq1840 showed that hydrolysis of NA6 or A5 but not NA4. This is probably because at least five-sugar units are essential for substrate recognition like ZgAgaC. Although the recombinant Aq1840 could cleave the β-1,4-linkage between A3 and NA2 of A5, it showed greater efficiency on the longer substrates such as NA6 or agarose. This result shows that the degradation by Aq1840 can be influenced by presence of extra sugars in addition to the five-sugar units.

Meanwhile, during the hydrolysis of NA6 by recombinant Aq1840, the substrate seemed to polymerize into larger oligosaccharides, but this polymerization was not observed in other reactions using smaller substrates. Even in several repeated tests, this type of polymerization still occurred (Fig. S6). Similarly, a previous study reported that a GH16 family β-agarase derived from the *Microbulbifer* sp. BN3 presented polymerization of oligosaccharides at the initial stage of NA8 hydrolysis (Li et al. 2020). Similar to this result, recombinant Aq1840 also exhibited polymerization properties, and the end-products of agarose were larger than NA4 until 72 h.

Just as genomic studies showed that marine Bacteroidetes may adapt to their habitat, analyses of the human gut metagenome and gut bacteria genomes revealed the presence of genes encoding porphyranases and agarases. These genes may have been laterally acquired from marine bacteria associated with seaweeds, and their products may function to decompose agarose or porphyran to oligo- or mono-saccharides (Pluvinage et al. 2018; Hehemann et al. 2010; Yun et al. 2021; Robb et al. 2022). The oligosaccharides could stimulate the growth of some probiotic bacteria, such as bifidobacteria and lactobacilli; thus, hydrolysates are considered to have prebiotic effects (Hu et al. 2006). As prebiotics, NAOSs (DPs of 4–6) increase the lactobacilli population, but larger NAOSs (DPs of 8–12) have better effects (Hu et al. 2006). Additionally, NAOS mixtures produced by treatment with β-agarase derived from *Vibrio natriegens* CICC 23,820 have the potential to be utilized as antioxidant and prebiotic additives (Zhang et al. 2019). The enzymatic characterization showed that recombinant Aq1840 could be used to produce the variable sizes of NAOS or AOS, and also the oligosaccharides can be utilized as prebiotic and antioxidant additives because of their large sizes.

In summary, we showed through whole-genome sequencing analysis that the genome of the ERC-38 strain contains all genes required for agarose degradation and metabolism and it can perform degradation at least via a functional β-agarase Aq1840. The result of minimum hydrolysis substrate test has shown that Aq1840 cleaves the β-1,4-linkage between A3 and NA2, which implies that the substrate recognition way of Aq1840 is identical to that of ZgAgaC. Characterization of Aq1840 supports the understanding of the action of relatively unique ZaAgaC-like enzymes and may facilitate industrial application of this recombinant enzyme for the production of oligosaccharides for use as food additives such as prebiotics or antioxidants. Moreover, the here presented in silico genomic analysis provides the gene basis for understanding the mechanism of marine polysaccharide degradation by *Aquimarina* sp. ERC-38.

## Supplementary Information

Below is the link to the electronic supplementary material.

Supplementary file1 (PDF 678 KB)

## Data Availability

The genome sequence data of the ERC-38 strain is available in the GenBank database (CP098511). Growth test data using carrageenan can be shared by request. Other data acquired and analyzed in this study are included in this paper and the supplementary information file.
