# Supplementary material for: Agarolytic Pathway in the Newly Isolated Aquimarina sp. Bacterial Strain ERC-38 and Characterization of a Putative β-agarase
Source: Mar Biotechnol (NY). 2023 Apr 1;25(2):314–27. doi: 10.1007/s10126-023-10206-7 (PMC10163077; doi:10.1007/s10126-023-10206-7)
Supplement: Supplementary file 1 — Supplementary file1 (PDF 678 KB) [file 10126_2023_10206_MOESM1_ESM.pdf]

Agarolytic pathway in the newly isolated *Aquimarina* sp. bacterial strain ERC-38 and characterization of a putative β-agarase.

Ji Young Kang[1,*], Ha-Yeon Song[2], and Jung-Mi Kim[2,*]

[1]Industrial Microbiology and Bioprocess Research Center, Korea Research Institute of Bioscience and Biotechnology (KRIBB), Jeongeup, Jeonbuk 56212, Republic of Korea
[2]Department of Bio-Environmental Chemistry, Institute of Life Science and Natural Resources, Wonkwang University, Iksan, Jeonbuk 54538, Republic of Korea

[*] Corresponding author:
Ji Young Kang
Tel.: +82 63 570 5118. Fax.: +82 63 570 5109. E-mail: jiyoka@kribb.re.kr
Jung-Mi Kim
Tel.: +82 63 850 6676. Fax.: +82 63 850 7308. E-mail: micro@wku.ac.kr

Table S1. Primer sequences used in this study.

Gene name	Primer names	Sequence	Product size (bp)	Annealing temperature (℃)
Aq1828	ERC-38_1828_F	5’-GAAGGAGATATACATATGGCTCAAGTAGAAGTGGAT-3’	2196	67
	ERC-38_1828_R	5’-GTGGTGGTGGTGCTCGAGCAACTTTACTATCTTTCT-3’		
Aq1829	ERC-38_1829_F	5’-GAAGGAGATATACATATGCAGGATTGGAAGGGAATT-3’	2658	68
	ERC-38_1829_R	5’-GTGGTGGTGGTGCTCGAGATTTTTAATAAGCTTTAG-3’		
Aq1830	ERC-38_1830_F	5’-GAAGGAGATATACATATGCAAAATGTTGAGGTCGAT-3’	3072	67
	ERC-38_1830_R	5’-GTGGTGGTGGTGCTCGAGTTTTTTAACTAATTTTAA-3’		
Aq1832	ERC-38_1832_F	5’-GAAGGAGATATACATATGTGCGAGCAAGTAGATGAA-3’	1110	68
	ERC-38_1832_R	5’-GTGGTGGTGGTGCTCGAGTTTACACGCTCGTTTATT-3’		
Aq1839	ERC-38_1839_F	5’-GAAGGAGATATACATATGCAGGTGAATGTTGATATT-3’	2595	66
	ERC-38_1839_R	5’-GTGGTGGTGGTGCTCGAGATACTTTACGAACTTAAT-3’		
Aq1830	ERC-38_1840_F	5’-GAAGGAGATATACATATGTGCGGAAGTGACGATCC-3’	1236	69
	ERC-38_1840_R	5’-GTGGTGGTGGTGCTCGAGGATATCCTCTAATTTCC-3’		

Supplementary Figure legends

Fig. S1. Growth characterization of *Aquimarina* sp. ERC-38 on marine medium. (a) The concave surface on a marine agar plate around an *Aquimarina* sp. ERC-38 colony (upper image). The lower images show a colony of this strain cultured on a 2% agar plate (left) and the transparent zone around the colony after staining with iodine (right). (b) The strain was cultured for 3 days at 25 °C in marine broth only or broth containing an additional carbon source such as glucose, galactose, or agarose.

Fig. S2. PUL involved in other types of marine polysaccharide metabolism in the ERC-38 genome. Each arrow indicates a separate gene, and the size is proportional to the gene length. Predicted functions are color-coded and presented above the arrows.

Fig. S3. Tentative pathway of carrageenan metabolism in the ERC-38 genome. Enzymes are indicated at each reaction step along with the CAZy family or EC number. Solid arrows represent the proposed ERC-38 pathway based on functional and bioinformatic analyses, and dotted arrows represent an unclear or absent pathway.

Fig. S4. Phylogenetic relationships of β-agarases of ERC-38 and other functionally validated β-agarases. Bootstrap percentages are based on 1,000 replicates; only values greater than 50% are shown at the nodes. Bar, 0.01 substitutions per nucleotide position.

Fig. S5. Agarose activity measurements and SDS-PAGE images of crude recombinant β-agarases. (a) Plate assay performed using the crude lysate of *E. coli* transformants containing the β-agarase expressing vector. A transparent zone is present on the crude lysate corresponding to Aq1829 and Aq1840. (b) Recombinant β-agarases (Aq1828, Aq1829, Aq1830, Aq1832, Aq1839, and Aq1840) were visualized by SDS-PAGE analysis. Control indicates proteins obtained from *E. coli* transformants containing an empty pET21a vector. Triangles indicate expressed recombinant proteins.

Fig. S6. Time-kinetic analysis of Aq1840-catalyzed hydrolysates of agarose, NAOS, and AOS as determined by TLC. For hydrolysis of each substrate, 1% NA6 and A5 were incubated with purified recombinant Aq1840 in 40 mM Tris-HCl buffer containing 30 mM NaCl and 1 mM DTT (pH 8.0) at 40 °C for the indicated time. The hydrolysates were spotted on silica gel 60 aluminum plates and developed with n-butanol:acetic acid:water (2:1:1, v/v) (a) or butanol:ethanol:water (3:2:2, v/v) (b and c). Saccharides were detected using a visualization solution containing 10% (v/v) sulfuric acid in ethanol, followed by heating to 100 °C.

Fig. S1

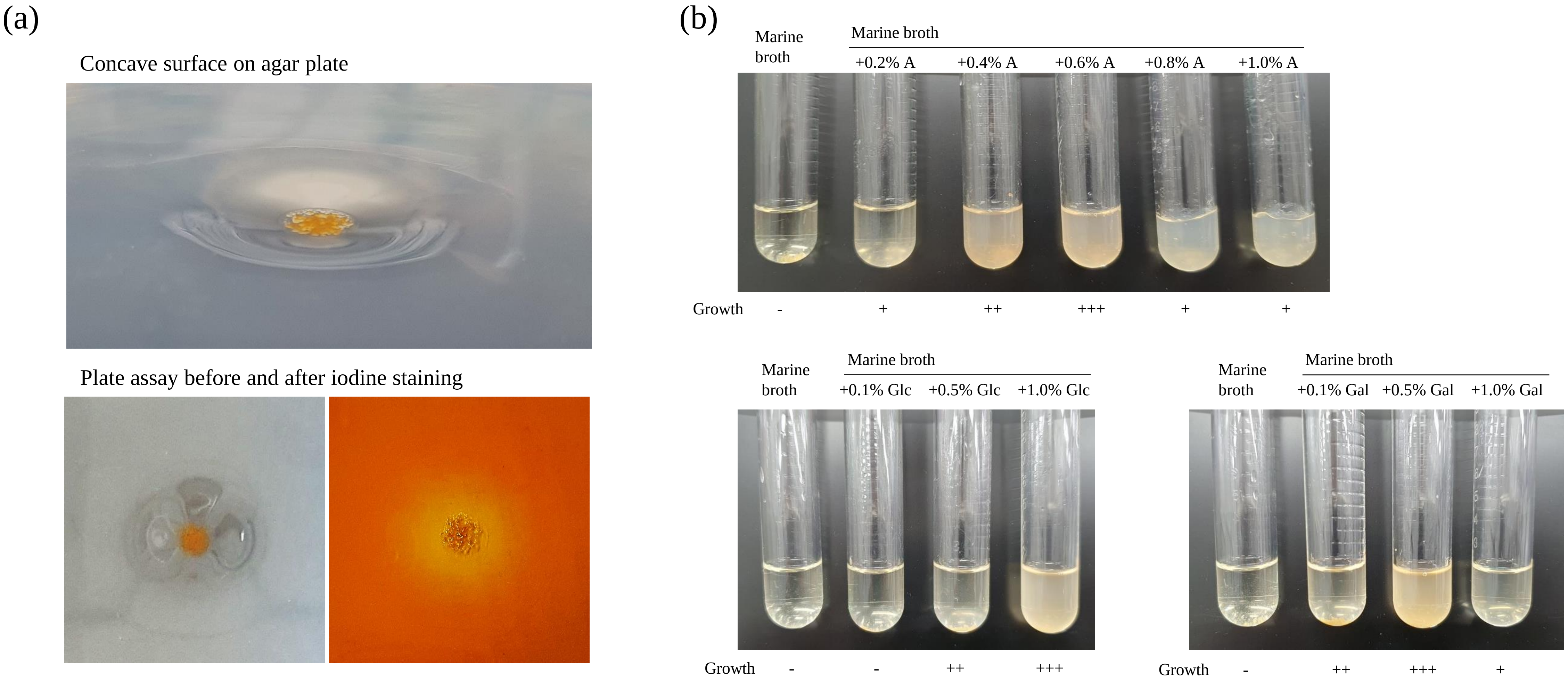
(a)
Concave surface on agar plate
Plate assay before and after iodine staining
(b)
Marine broth
Marine broth
+0.2% A
+0.4% A
+0.6% A
+0.8% A
+1.0% A
Growth - + ++ +++ + +
Marine broth
Marine broth
+0.1% Glc
+0.5% Glc
+1.0% Glc
Growth - - ++ +++
Marine broth
Marine broth
+0.1% Gal
+0.5% Gal
+1.0% Gal
Growth - ++ +++ +

Fig. S2

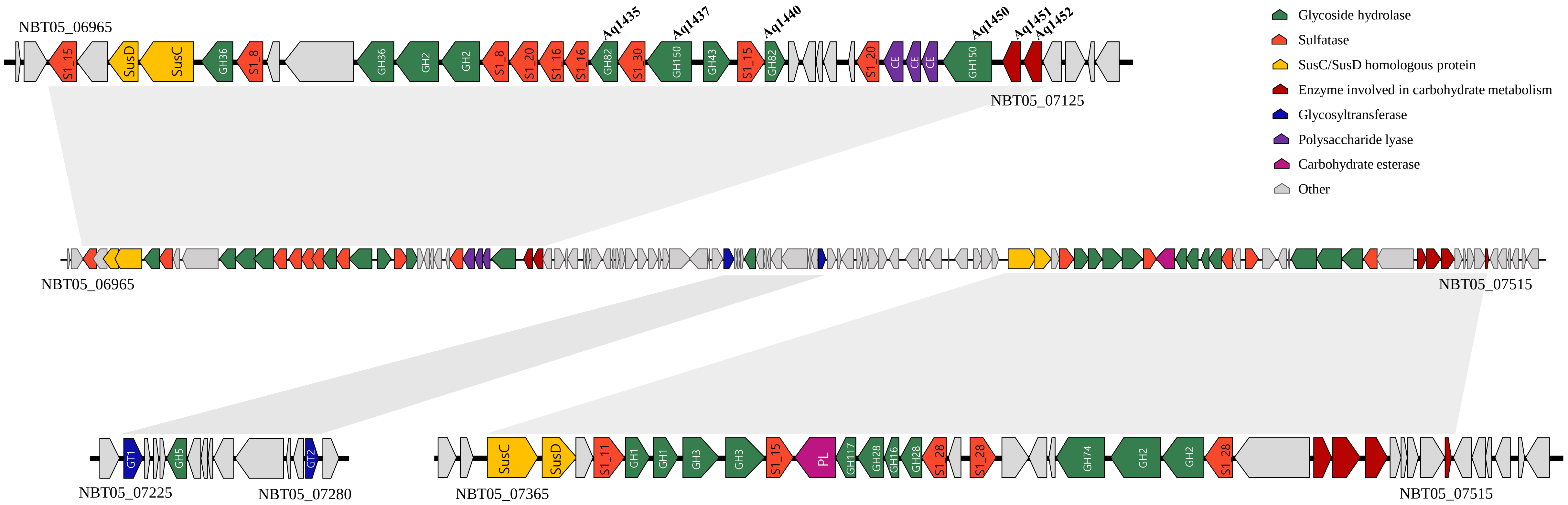

NBT05_06965
Aq1435
Aq1437
Aq1440
Aq1450
Aq1451
Aq1452
NBT05_07125
NBT05_06965
NBT05_07515
NBT05_07225
NBT05_07280
NBT05_07365
NBT05_07515
Glycoside hydrolase
Sulfatase
SusC/SusD homologous protein
Enzyme involved in carbohydrate metabolism
Glycosyltransferase
Polysaccharide lyase
Carbohydrate esterase
Other

Fig. S3

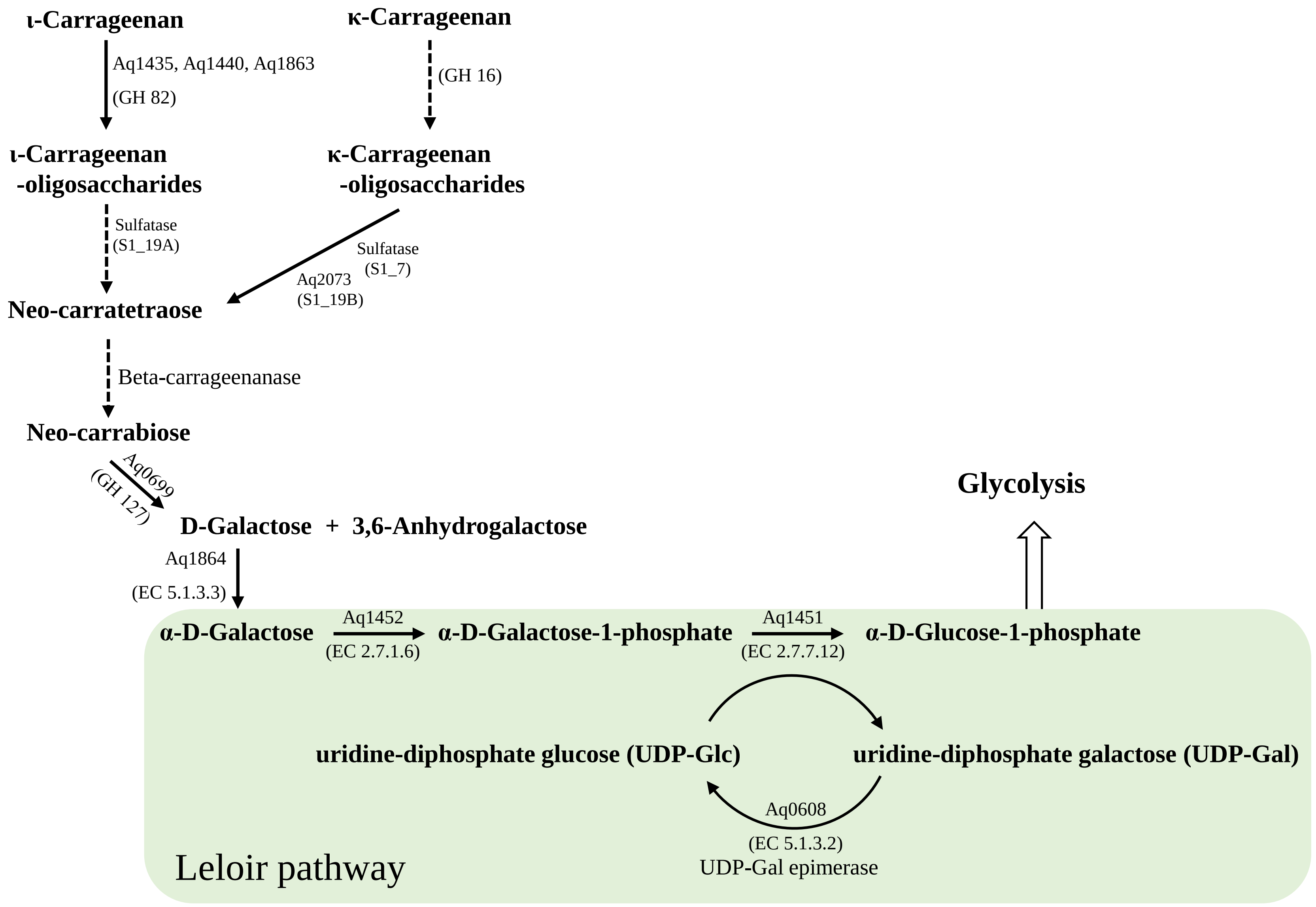

ι-Carrageenan
κ-Carrageenan
Aq1435, Aq1440, Aq1863
(GH 82)
(GH 16)
ι-Carrageenan
-oligosaccharides
κ-Carrageenan
-oligosaccharides
Sulfatase
(S1_19A)
Sulfatase
(S1_7)
Aq2073
(S1_19B)
Neo-carratetraose
Beta-carrageenanase
Neo-carrabiose
Aq0699
(GH 127)
D-Galactose + 3,6-Anhydrogalactose
Glycolysis
Aq1864
(EC 5.1.3.3)
α-D-Galactose
Aq1452
(EC 2.7.1.6)
α-D-Galactose-1-phosphate
Aq1451
(EC 2.7.7.12)
α-D-Glucose-1-phosphate
uridine-diphosphate glucose (UDP-Glc)
uridine-diphosphate galactose (UDP-Gal)
Aq0608
(EC 5.1.3.2)
UDP-Gal epimerase
Leloir pathway

Fig. S4

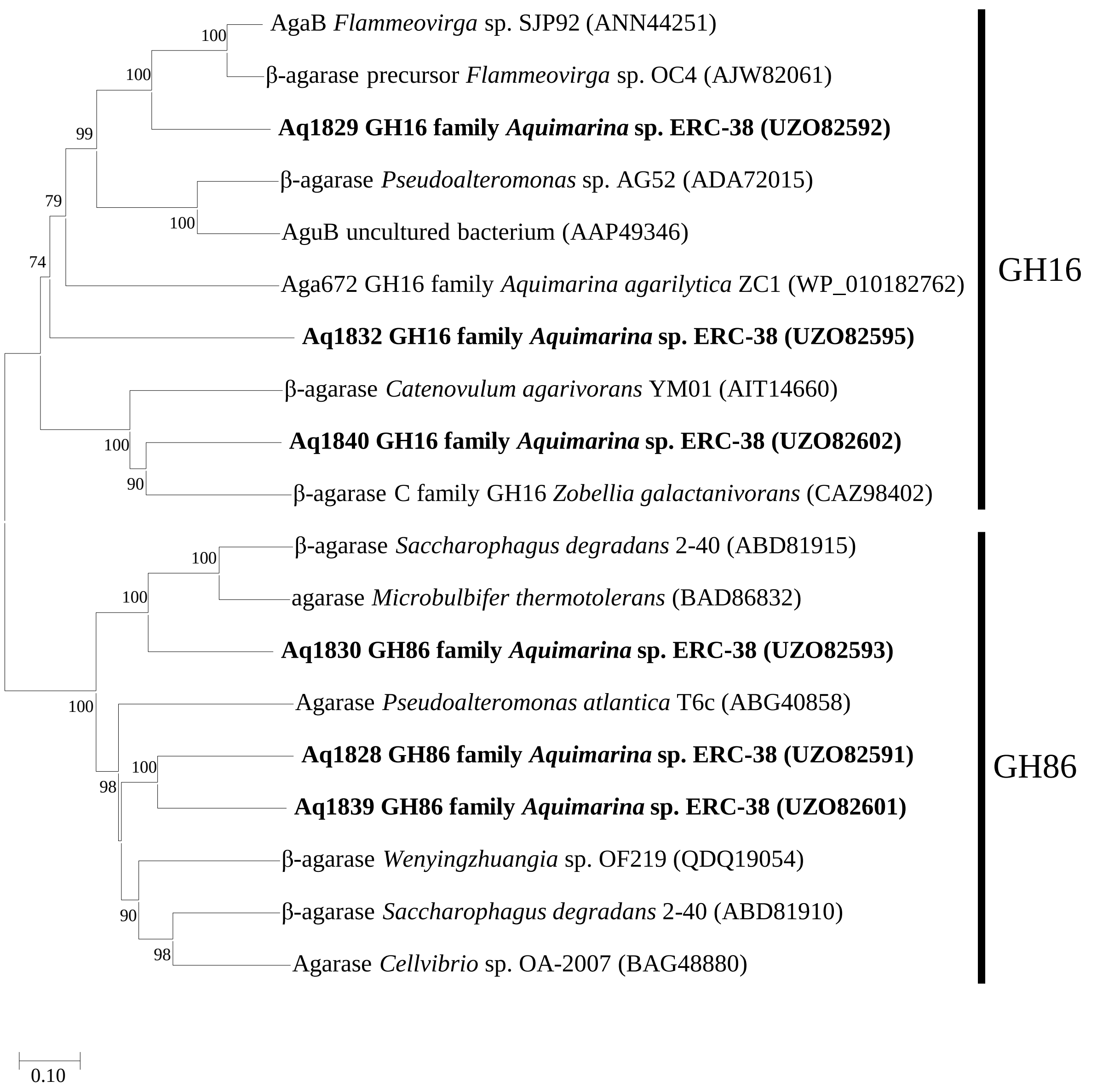

AgaB Flammeovirga sp. SJP92 (ANN44251)
β-agarase precursor Flammeovirga sp. OC4 (AJW82061)
Aq1829 GH16 family Aquimarina sp. ERC-38 (UZO82592)
β-agarase Pseudoalteromonas sp. AG52 (ADA72015)
AguB uncultured bacterium (AAP49346)
Aga672 GH16 family Aquimarina agarilytica ZC1 (WP_010182762)
Aq1832 GH16 family Aquimarina sp. ERC-38 (UZO82595)
β-agarase Catenovulum agarivorans YM01 (AIT14660)
Aq1840 GH16 family Aquimarina sp. ERC-38 (UZO82602)
β-agarase C family GH16 Zobellia galactanivorans (CAZ98402)
β-agarase Saccharophagus degradans 2-40 (ABD81915)
agarase Microbulbifer thermotolerans (BAD86832)
Aq1830 GH86 family Aquimarina sp. ERC-38 (UZO82593)
Agarase Pseudoalteromonas atlantica T6c (ABG40858)
Aq1828 GH86 family Aquimarina sp. ERC-38 (UZO82591)
Aq1839 GH86 family Aquimarina sp. ERC-38 (UZO82601)
β-agarase Wenyingzhuangia sp. OF219 (QDQ19054)
β-agarase Saccharophagus degradans 2-40 (ABD81910)
Agarase Cellvibrio sp. OA-2007 (BAG48880)
100
100
99
100
79
74
100
90
100
100
100
98
100
90
98
GH16
GH86
0.10

Fig. S5

(a)

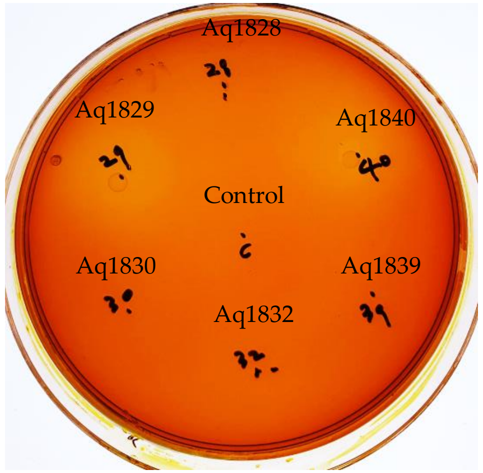

Aq1828
Aq1829
Aq1840
Control
Aq1830
Aq1839
Aq1832

(b)

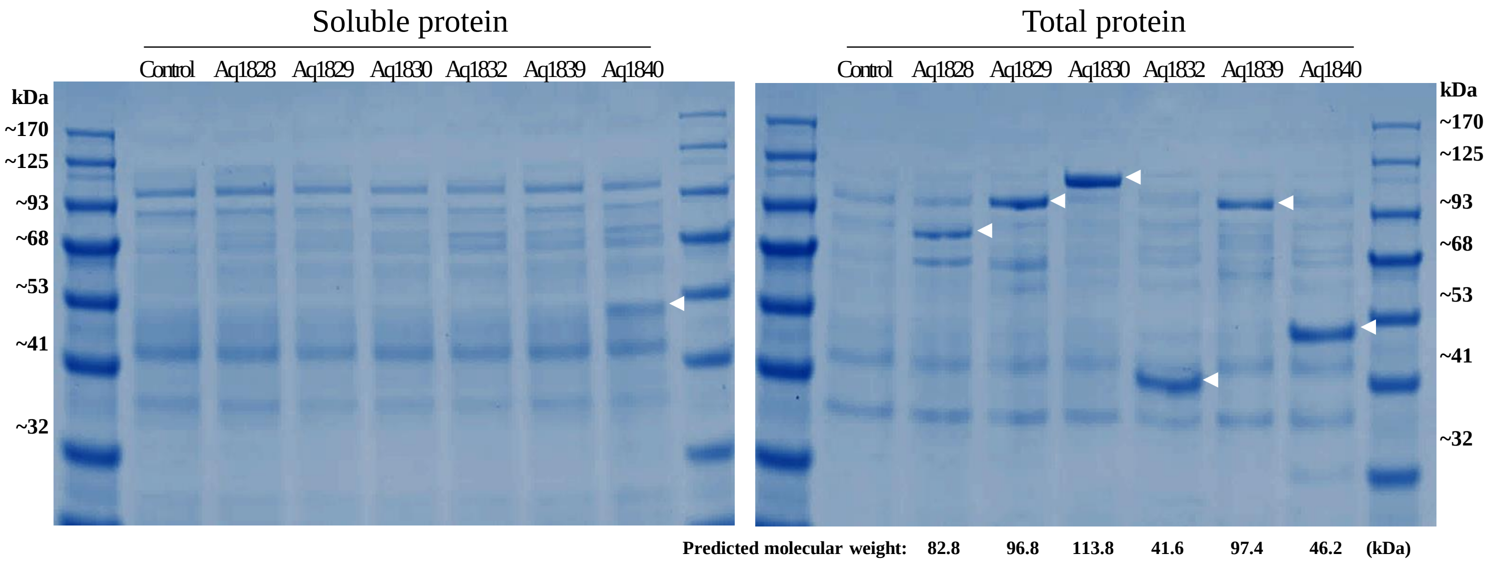

Soluble protein
Total protein
Control
Aq1828
Aq1829
Aq1830
Aq1832
Aq1839
Aq1840
kDa
~170
~125
~93
~68
~53
~41
~32
Predicted molecular weight: 82.8 96.8 113.8 41.6 97.4 46.2 (kDa)

Fig. S6

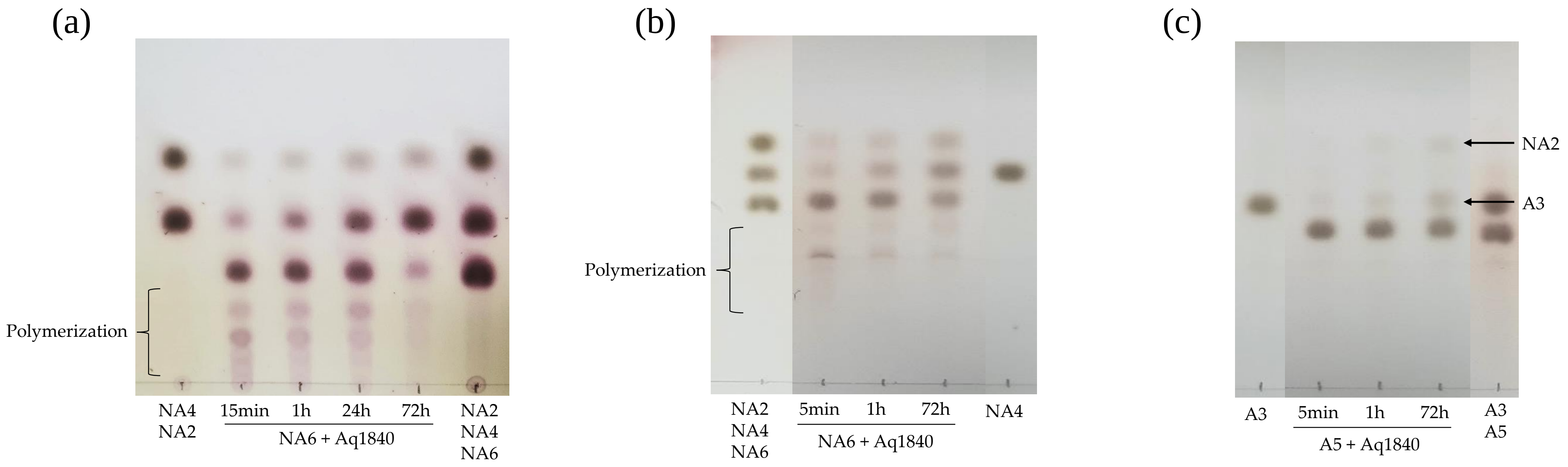

(a)
Polymerization
NA4
NA2
15min
1h
24h
72h
NA6 + Aq1840
NA2
NA4
NA6
(b)
Polymerization
NA2
NA4
NA6
5min
1h
72h
NA6 + Aq1840
NA4
(c)
NA2
A3
A3
5min
1h
72h
A5 + Aq1840
A3
A5
